# Resveratrol fuels HER2 and ERα-positive breast cancer behaving as proteasome inhibitor

**DOI:** 10.18632/aging.101175

**Published:** 2017-02-26

**Authors:** Cristina Andreani, Caterina Bartolacci, Kathleen Wijnant, Rita Crinelli, Marzia Bianchi, Mauro Magnani, Albana Hysi, Manuela Iezzi, Augusto Amici, Cristina Marchini

**Affiliations:** ^1^ School of Biosciences and Veterinary Medicine, University of Camerino, Camerino, 62032, Italy; ^2^ Department of Biomolecular Sciences, Section of Biochemistry and Molecular Biology, University of Urbino “Carlo Bo”, Urbino, 61029, Italy; ^3^ Aging Research Centre, G. d'Annunzio University, Chieti, 66100, Italy

**Keywords:** breast cancer, Δ16HER2 mice, resveratrol, estrogen receptor, proteasome

## Abstract

The phytoestrogen resveratrol has been reported to possess cancer chemo-preventive activity on the basis of its effects on tumor cell lines and xenograft or carcinogen-inducible *in vivo* models. Here we investigated the effects of resveratrol on spontaneous mammary carcinogenesis using Δ16HER2 mice as HER2+/ERα+ breast cancer model. Instead of inhibiting tumor growth, resveratrol treatment (0.0001% in drinking water; daily intake of 4μg/mouse) shortened tumor latency and enhanced tumor multiplicity in Δ16HER2 mice. This *in vivo* tumor-promoting effect of resveratrol was associated with up-regulation of Δ16HER2 and down-regulation of ERα protein levels and was recapitulated *in vitro* by murine (CAM6) and human (BT474) tumor cell lines. Our results demonstrate that resveratrol, acting as a proteasome inhibitor, leads to Δ16HER2 accumulation which favors the formation of Δ16HER2/HER3 heterodimers. The consequential activation of downstream mTORC1/p70S6K/4EBP1 pathway triggers cancer growth and proliferation. This study provides evidence that resveratrol mechanism of action (and hence its effects) depends on the intrinsic molecular properties of the cancer model under investigation, exerting a tumor-promoting effect in luminal B breast cancer subtype models.

## INTRODUCTION

Several natural compounds have recently gathered renewed interest as potential anti-tumor agents. Resveratrol (*trans* -3,4′,5-trihydroxystilbene), a natural polyphenol found in grapes, peanuts, cocoa, berries, and red wine, has been described as a putative cancer chemo-preventive compound able to counteract breast cancer initiation, promotion and progression [1]. Although several *in vitro* studies have proposed resveratrol to exert an anti-tumor activity by inducing apoptosis and/or cell cycle arrest in various mammary cancer cell lines [2-4], investigations attempting to dissect the *in vivo* effects of resveratrol upon cancer onset and progression have led to contradictory results [5-7]. Such a controversial scenario can be explained recalling that resveratrol, as a phytoestrogen, possesses both estrogenic and anti-estrogenic activities on ERɑ-positive breast cancer [8-10]. The devised experimental conditions -dose, timing, way of administration- influence the outcome of resveratrol-based studies as well [11]. Moreover, the experimental model taken into consideration is determinant: the majority of available data has been obtained using transplantable or carcinogen-inducible tumor models that, even if infor-mative, fail to reproduce the complexity of spontaneous tumorigenesis [11]. To our knowledge, the solely *in vivo* study on a spontaneous mammary tumor model was reported by Provinciali et al. who claimed how resveratrol supplementation delays tumor onset, growth and metastases in HER2/neu transgenic female mice [12]. This research was performed *in vivo* on transgenic mice carrying the rat HER2/neu oncogene under the transcriptional control of MMTV promoter, and *in vitro* on human SKBR3 cell line, both models representing ER-negative (-) and HER2-positive (+) breast cancers [13]. They ascribed such protective effects to a decrease in HER2 gene expression and a promotion of *in situ* apoptosis observed inside mammary tumor tissues [12].

Even though HER2 over-expression is considered a hallmark of aggressive breast cancer subtypes, increasing evidence has pointed toward a major contribution of Δ16HER2 splice variant (lacking exon-16), which is commonly co-expressed with wild-type HER2, in promoting cancer progression, metastasis and resistance to Trastuzumab [14, 15]. The relevance of Δ16HER2 and the availability of Δ16HER2 mice, transgenic for the human Δ16HER2 oncogenic isoform, drove us to evaluate the effects of resveratrol intake on Δ16HER2-driven mammary carcinogenesis [16]. Unexpectedly, we found resveratrol to promote Δ16HER2 carcinogenesis by up-regulating Δ16HER2 protein levels in both primary tumors and metastases and down-regulating ERɑ expression in tumors, thus recapitulating endocrine therapy resistance. Mechanistically, resveratrol partially inhibits proteasome activity and leads to Δ16HER2 protein accumulation. Increased Δ16HER2 levels, in turn, trigger the formation of Δ16HER2/HER3 heterodimers and the consequent HER3 phosphorylation. The resulting activation of mTORC1/p70S6K pathway finally fuels cell proliferation and mammary tumor development in Δ16HER2 mice.

## RESULTS

### Resveratrol supplementation promotes mammary tumor development in Δ16HER2 mice

**Figure 1:**
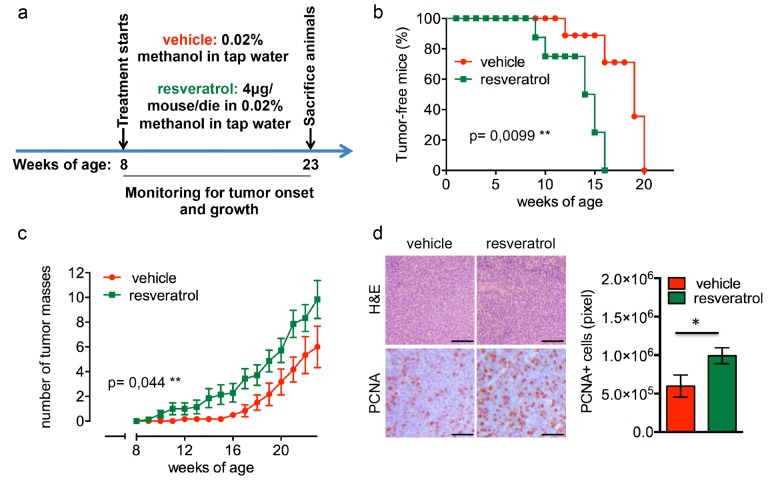
Resveratrol fuels mammary tumor formation in a luminal B breast cancer *in vivo* model (**a**) Schematic representation of the experimental workflow used for the treatment of Δ16HER2 mice with vehicle or resveratrol. (**b**) Kaplan-Meier disease-free survival plot for vehicle- (*n*= 9) and resveratrol-treated (*n*= 9) Δ16HER2 mice. **p ≤ 0.01, Log Rank test. (**c**) Tumor multiplicity in resveratrol-treated *vs* control mice; the number of palpable mammary tumors per mouse is represented as mean ± SD. Statistical significance was assessed by two-way ANOVA test. (**d**) Left panel: Representative Hematoxyilin-Eosin (H&E) and PCNA stained sections of tumors from resveratrol and vehicle treated mice. Magnification 400 X. Right panel: Quantification of PCNA staining in tumors from resveratrol and vehicle treated mice. Data are expressed as mean ± SEM. *p < 0.05; unpaired two-tailed student t test.

In Δ16HER2 mice, the MMTV-driven expression of only five copies of the human Δ16HER2 transgene triggers neoplastic transformation of mammary epithelial cells with a short latency time. Since all transgenic females develop multiple asynchronous mammary tumors on average at the 15^th^ week of age, resveratrol treatment started when the animals were 8 week-old and proceeded up to 23 weeks of age (Figure 1a). As shown in Figure 1b, resveratrol-receiving mice displayed a significantly anticipated tumor onset developing the first tumor mass at only 9 weeks of age (p=0.0099), while the first tumor appeared at 12 weeks of age in untreated females. In particular, 50% of resveratrol-treated mice presented at least one mammary mammary tumor at 15 weeks of age, when 90% of the control littermates were still tumor-free, although all the animals eventually developed tumors within 20 weeks of age. Moreover, as shown in Figure 1c, mice supplemented with resveratrol suffered from a higher number of tumor masses than controls, with an average of 9 tumors/mouse and 6 tumors/mouse, respectively (p=0.044). The enhanced proliferation rate triggered by resveratrol correlates with an increased expression of proliferating cell nuclear antigen (PCNA) in tumors from treated animals as demonstrated by immuno-histochemical (IHC) analysis (Figure 1d).

#### Resveratrol triggers Δ16HER2 over-expression and ERɑ down-regulation

**Figure 2:**
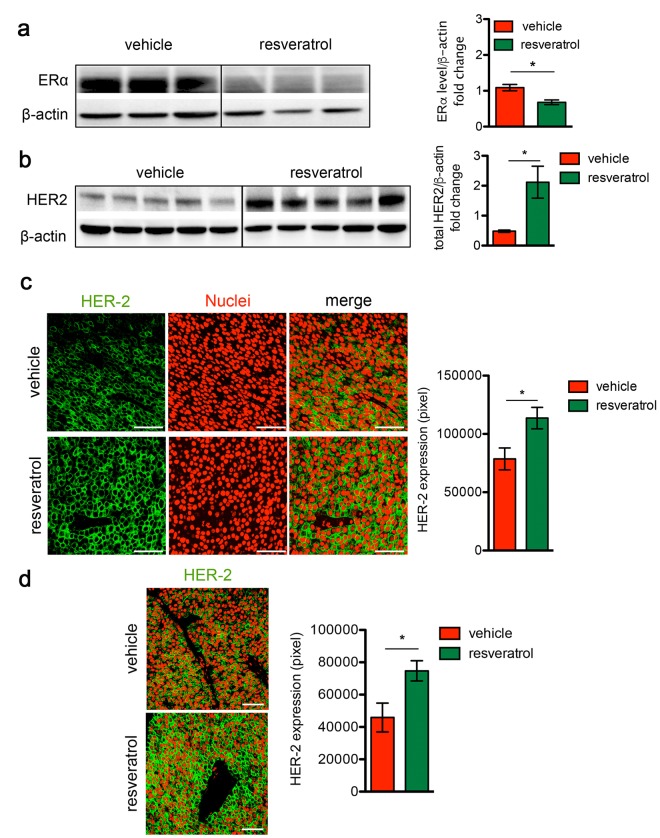
Resveratrol treatment induces HER2 over-expression and ERɑ down-regulation in HER2+/ERα+ mammary carcinomas (**a**) Representative western blot analysis of ERα and β-actin (loading control) in spontaneous mammary tumors from Δ16HER2 mice supplemented or not with resveratrol (left panel), and relative densitometry quantification from three independent experiments (right panel). The significance was determined by unpaired two-tailed student t test, *p < 0.05. (**b**) Representative western blot analysis of HER2 and β-actin (loading control) in spontaneous mammary tumors from Δ16HER2 mice, supplemented or not with resveratrol (left panel), and relative densitometry quantification from three independent experiments (right panel). The significance was determined by unpaired two-tailed student t test, *p < 0.05. (**c**) Left panel: representative immunofluorescence images of tumor sections from control and resveratrol supplemented mice stained with an antibody anti-HER2 (green) and DRAQ5 dye (red) for nuclei staining. Magnification 400 X. Right panel: quantification of HER2 staining in tumors from resveratrol and vehicle treated mice. Data are expressed as mean ± SEM. *p < 0.05; unpaired two-tailed student t test. (**d**) Resveratrol treatment induces HER2 over-expression in lung metastases. Left panel: representative immunofluorescence images of lung metastasis sections from control and resveratrol supplemented mice stained with an antibody anti-HER2 (green) and DRAQ5 (red). Magnification 400 X. Right panel: quantification of HER2 staining in tumors from resveratrol and vehicle treated mice. Data are expressed as mean ± SEM. *p < 0.05; unpaired two-tailed student t test.

To investigate the molecular mechanisms underlying the boosted cancer growth induced by resveratrol, ERɑ and Δ16HER2 protein levels were analyzed by western blot in tumors explanted from resveratrol-treated and control mice. We found that *in vivo* administration of resveratrol caused a dramatic drop in the ERɑ expression (Figure 2a, p<0.05) and a concomitant increase in Δ16HER2 protein level (Figure 2b, p<0.05) in tumors. Confocal micro-scopy analysis confirmed the significant higher abundance of Δ16HER2 in both primary tumors (Figure 2c) and lung metastatic lesions (Figure 2d, p<0.05) from resveratrol-treated mice compared to controls. These data suggest that the effect of resveratrol on Δ16HER2 protein accumulation is similar in primary tumors and metastases, although resveratrol supplementation did not seem to influence the number of lung metastases (3 out of 6 mice were found with pulmonary metastases in the ve-hicle group, 4 out of 7 mice in resveratrol-group, Fig. S1).

#### Resveratrol *in vitro* treatment of Δ16HER2+/ERα+ breast cancer cell lines (luminal B subtype) recapitulates the *in vivo* outcomes

To confirm the pro-tumor effect of resveratrol in human Δ16HER2+/ERα+ breast cancer, we tested resveratrol *in vitro* on human BT474 and murine CAM6 cell lines.

**Figure 3:**
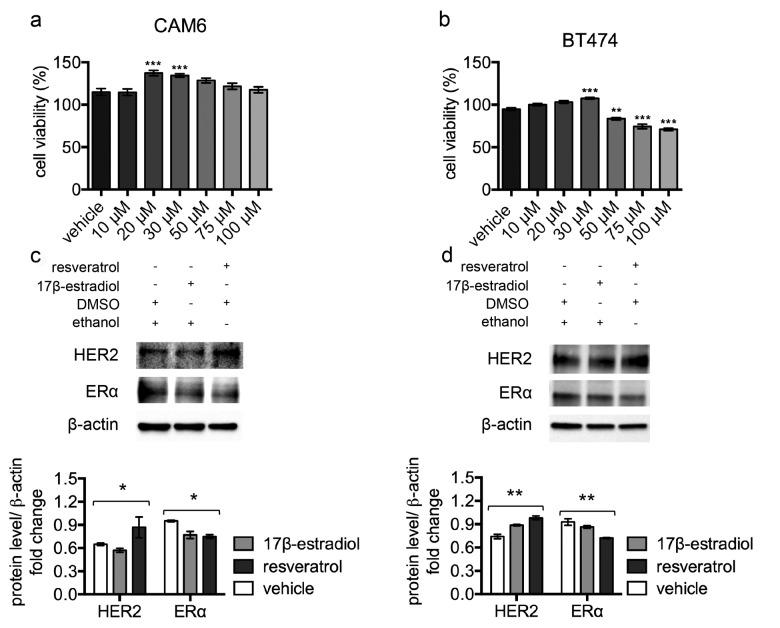
Resveratrol triggers HER2 over-expression and ERɑ down-regulation in luminal B breast cancer cell lines (**a**) CAM6 and (**b**) BT474 cells were incubated for 24 hours in the presence of vehicle or increasing concentrations of resveratrol and cell viability was determined by MTT assay. Results (including vehicle group treated with 0.02 % DMSO) are expressed as percentage (%) of cell viability relative to untreated controls. Columns, mean of three separate experiments wherein each treatment was repeated in 16 wells; bars, SE. **p ≤ 0.01, ***p ≤ 0.001, one-way ANOVA followed by Bonferroni's multiple comparison test. Representative western blot analysis of HER2, ERα and β-actin (loading control) in murine CAM6 cells (**c**) or human BT474 cells (**d**), treated with resveratrol or 17β-estradiol or vehicle for 24 hours (upper panel), and relative densitometry quantification (lower panel). The significance was determined by one-way ANOVA (*p < 0.05, **p ≤ 0.01).

CAM6 cells have been established from Δ16HER2 mice and can be considered the *in vitro* counterpart of Δ16HER2 mammary tumors [17]. After 24 hours’ incubation, despite high resveratrol concentrations resulted in reduced cell viability, low-range resveratrol concentrations (20-30 μM) promoted cell proliferation of both CAM6 and BT474 cells (Figure 3a and 3b), mimicking the *in vivo* resveratrol effect. Consistently, western blot analysis revealed that treatment with 40 μM resveratrol for 24 hours induced HER2 up-regulation and a concomitant ERɑ down-regulation in both CAM6 and BT474 cells, recapitulating the molecular events observed inside Δ16HER2 tumors (Figure 3c and 3d). Since 10 nM 17β-estradiol treatment only partially reproduced the resveratrol effect in CAM6 cells, inducing reduction of ERɑ without enhancing HER2 protein levels, we suppose that resveratrol exerts a more complex action on Δ16HER2+ cancer cells than the mere estrogen-like effect.

#### Resveratrol causes Δ16HER2 protein accumulation by partial inhibition of proteasome activity

**Figure 4:**
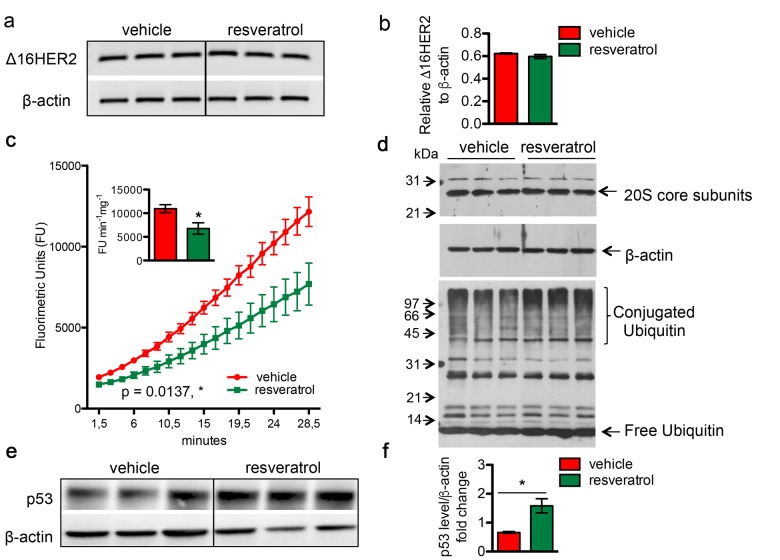
Resveratrol inhibits the chymotrypsin-like activity of 20S proteasome and resulted in an increased accumulation of protein-ubiquitin conjugates Δ16HER2 and β-actin (internal control) mRNA levels were measured by semi-quantitative RT-PCR (**a**) and qRT-PCR (**b**) analyses in spontaneous mammary tumors from Δ16HER2 mice supplemented or not with resveratrol. (**c**) The chymotrypsin-like activity of the 20S proteasome was measured in tumor samples, from Δ16HER2 mice treated or not with resveratrol, as described in Materials and Methods, and expressed as fluorimetric units (FU) min^-1^ mg^-1^. The significance was determined by unpaired two-tailed student t test, *p < 0.05. (**d**) Western blot analysis of 20S proteasome subunit content (upper panel) and ubiquitin-protein conjugates and free ubiquitin levels (lower panel) in tumor samples from Δ16HER2 mice treated or not with resveratrol. β-actin was used as loading control. (**e**) Representative western blot analysis of p53 and β-actin (loading control) in spontaneous mammary tumors from Δ16HER2 mice, supplemented or not with resveratrol, and (**f**) relative densitometric quantification from three independent experiments. The significance was determined by unpaired two-tailed student t test, *p < 0.05.

To evaluate whether the resveratrol-induced Δ16HER2 over-expression was due to transcriptional or post-transcriptional mechanisms, Δ16HER2 mRNA levels were analyzed by qRT-PCR in mammary tumors from resveratrol-treated and control mice. Despite the significantly higher level of Δ16HER2 protein detected in resveratrol-treated tumors in comparison with controls (Figure 2b and 2c), no differences in Δ16HER2 mRNA levels were found between the two experimental groups (Figure 4a and 4b). By contrast, the activity of the 20S proteasome, which is responsible for the degradation of the bulk of the cellular proteins, was significantly affected by *in vivo* supplementation of resveratrol, as determined by the 20S chymotrypsin-like activity assay in tumor extracts (Figure 4c). Noteworthy, no difference was found in the cellular levels of the 20S subunits, suggesting that resveratrol may exert a direct inhibitory effect on the proteasome enzymatic activity (Figure 4d, upper panel). In agreement with a partial inhibition of the proteasome, tumor masses from resveratrol-treated mice displayed a higher extent of ubiquitin-conjugated proteins with respect to those obtained from mice receiving placebo (Figure 4d, lower panel). Consistently, western blot analysis also revealed a significant increase in the levels of p53 protein in mammary tumors from resveratrol-receiving mice in comparison with controls (Figure 4e and 4f, p<0.05), giving further evidence of resveratrol-mediated proteasome inhibition. Noteworthy, such p53 augmentation inside tumors of resveratrol group did not trigger a concomitant up-regulation in the apoptotic cell death, as indicated by IHC analysis of cleaved caspase 3 (Figure S2). Moreover, the ability of resveratrol to inhibit in a concentration-dependent manner the activity of the proteasome was confirmed in both BT474 and CAM6 cell extracts (Figure S3).

#### Δ16HER2 accumulation induced by resveratrol results in a preferential activation of mTORC1/p70S6K/4EBP1 pathway

**Figure 5:**
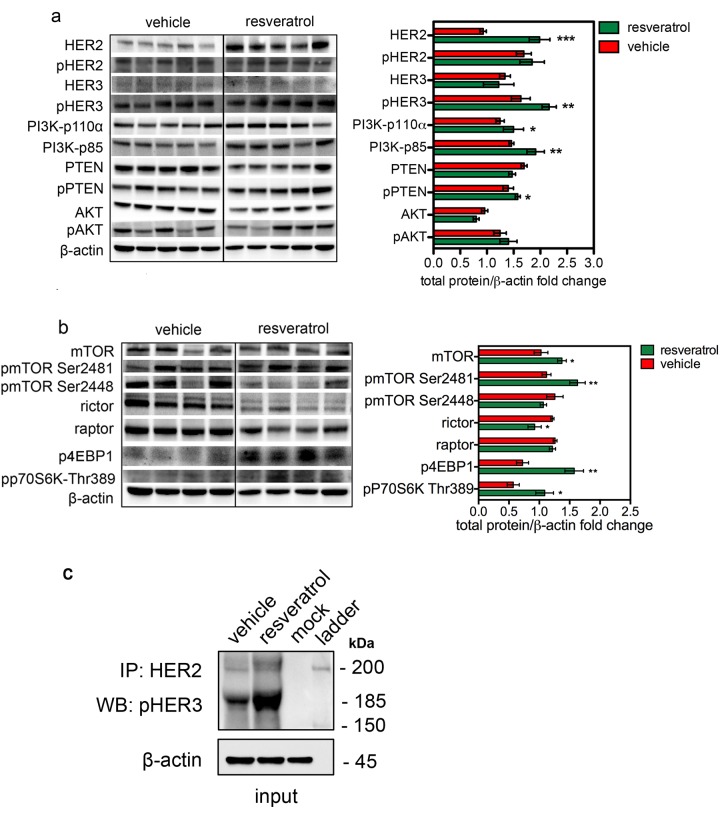
Increased HER2 level induced by resveratrol results in a preferential activation of mTORC1/p70S6K pathway Representative western blot analysis of HER2 and HER3 downstream signaling pathways in spontaneous mammary tumors from Δ16HER2 mice, treated or not with resveratrol (left panels), and densitometry quantification from three independent experiments (right panels). (**a**) PI3K/AKT and (**b**) mTOR signaling pathways were analyzed. β-actin was used as loading control. The significance was determined by t-test (*p < 0.05; **p ≤ 0.01; ***p ≤ 0.001). (**c**) Resveratrol promotes the direct coupling of Δ16HER2 to HER3 kinase in HER2+/ERα+ breast cancer. Δ16HER2 kinase co-immunoprecipitates with pHER3. Proteins were immunoprecipitated with anti-HER2 antibody and then probed by western blot for pHER3. Input represents 10% of the co-immunoprecipitation protein amount (30 μg).

To identify the signaling cascades beneath the resveratrol-induced Δ16HER2 accumulation, the main oncogenic pathways known to be involved in breast carcinogenesis were analyzed by western blot. As shown in Figure 5a, despite tumors from resveratrol-treated mice expressed significantly higher Δ16HER2 levels than vehicle counterparts, no relevant differences were detected in its phosphorylation status. Noteworthy, phosphorylated HER3 levels were significantly higher in tumor extracts from resveratrol-treated animals (Figure 5a). Consistent with the over-expression of Δ16HER2 and increased HER3 phosphorylation, tumors displayed high levels of Δ16HER2-HER3 heterodimer upon resveratrol treatment, as measured by co-immunoprecipitation assay (Figure 5c). This result suggests that resveratrol-induced accumulation of Δ16HER2 leads to Δ16HER2/HER3 heterodimer formation and consequently to HER3 transactivation. It is known that HER2/HER3 heterodimers are powerful oncogenic units, in part because phospho-HER3 boosts PI3K-AKT-mTOR signaling pathway [18].

Consistently, we found that resveratrol-induced Δ16HER2/HER3 heterodimerization caused a concomitant increase in the levels of PI3K p85 and PI3K p110ɑ (PI3K-p85, p<0.01 and PI3K-p110ɑ, p<0.05) and the up-regulation of the mammalian target of rapamycin complex 1 (mTORC1). The mTOR Ser/Thr kinase is the catalytic component of two structurally and functionally distinct signaling complexes. The rictor-containing mTORC2 regulates the actin cytoskeleton and activates Akt [19]. The raptor-containing mTORC1 regulates cell growth and nutrient signaling [20]. In particular, mTORC1 phosphorylates p70 S6 kinase (S6K) and eukaryotic initiation factor 4E binding protein 1 (4EBP1), thereby regulating translation of proteins that are critical for progression from G1 into S phase [21]. Therefore, we examined levels of phospho-4EBP1 and phospho-S6K as readouts of mTORC1 activity. As shown in Figure 5b, resveratrol significantly increased both phospho-4EBP1 (p<0.01) and phospho-S6K levels (p<0.05) relative to vehicle. The phosphorylation of these two *par excellence* mTORC1 downstream effectors positively correlated with the level of mTOR Ser(P)-2481, while lower rictor and mTOR Ser(P)-2448 pointed toward inhibition of mTORC2. Interestingly, although resveratrol supplementation promoted the inhibiting phoshorylation of PTEN (pPTEN ser380/thr382/383) [22], no differences in terms of AKT activation (pAKT) were observed between resveratrol and control groups: this may be due to feedback loops that compensate PI3K-mediated AKT activation. Interestingly, other pathways known to be activated downstream Δ16HER2, such as the Δ16HER2/Src/STAT3 axis, did not vary significantly between the two experimental groups (Figure S4). It is worth to note that the levels of unphosphorylated proteins other than HER2, like mTOR (Figure 5b), Src and Erk1,2 (Figure S4) increased in tumor extracts from resveratrol group. This observation is consistent with the ubiquitin-proteasome system (UPS)-dependent degradation of these proteins [23-26] and supports our evidence about resveratrol acting as proteasome inhibitor.

### DISCUSSION

The need for safe and low-toxic preventive and therapeutic strategies against cancer is still unmet. In this scenario phytoestrogens like resveratrol have received growing attention as valuable chemo-preventive tools. Contrarily to previously published results [12], describing an antitumor effect of resveratrol in a HER2+/ERα- breast cancer experimental model, here we have found that resveratrol, given at the same reported dose, regimen and administration way, did not prevent nor delay tumor onset; instead, significantly accelerated cancer development in Δ16HER2 females that spontaneously develop HER2+/ERα+ mammary carcinomas.

In Δ16HER2 mice, resveratrol intake not only anticipated the tumor onset, but significantly augmented tumor multiplicity, promoting the growth of a higher number of tumor masses per mouse. Moreover, consistently with its pro-proliferative effect, we provide evidence that resveratrol treatment induced a strong increase in Δ16HER2 expression, associated with a significant reduction of ERα protein level. In contrast to the observation by Provinciali et al., resveratrol did not affect Δ16HER2 mRNA levels, suggesting that Δ16HER2 protein accumulation was the consequence of a reduced degradation rather than an increased transcription. Indeed, it has been demonstrated that resveratrol can act as a potent proteasome inhibitor [27, 28]. In our experimental settings, resveratrol treatment partially inhibited the chymotrypsin-like proteasome activity, which may elicit the observed accumulation of Δ16HER2 protein in mammary tumors, that in turn fuels cancer growth. Noteworthy, the same result was obtained *in vitro* using murine and human luminal B breast cancer cell lines (CAM6 and BT474 cells, respectively). Indeed, adding resveratrol to cell-free extracts of CAM6 and BT474 resulted in a concentration-dependent proteasome inhibition. On the whole, the *in vivo* and *in vitro* evidence we gathered suggests that resveratrol can act as a proteasome inhibitor as Qureshi et al. claimed [26]. Nonetheless, we cannot exclude that additional effects of resveratrol on signaling cascades might eventually mediate an indirect proteasome inhibition as well. Anyhow, the ability of resveratrol to inhibit the proteasome can be considered a double-edged sword. On one side, resveratrol has been reported to exert anti-inflammatory effects partially by counteracting the proteasome-mediated activation of NF-κB, thereby suppressing activation of pro-inflammatory cytokines and iNOS genes [26]. Since inflammation is a hallmark of neurodegenerative disorders and aging [29, 30], proteasome inhibition triggered by resveratrol might provide additional insights into its anti-aging properties as well. On the other side, as our evidence has demonstrated, proteasome impairment and the consequent HER2 accumulation can promote breast cancer development. Strikingly, this resveratrol-induced Δ16HER2 increase was associated with a concomitant ERα diminution. ERα is of critical importance in mammary cancer initiation and progression, having become an ideal target for anti-cancer therapies. However, expression of ERα in tumors is dynamic and can change during the course of tumor progression and following therapy [31, 32]. Loss of ERα represents a crucial mechanism for the acquisition of endocrine resistance [33-35]. Several clinical and preclinical studies have confirmed the existence of a remarkable crosstalk between HER2 and ERα that usually leads them to be inversely expressed inside breast cancer. Strikingly, such bidirectional fluctuations dictate the responsiveness to HER2- and/or ER-targeted therapies [36-38]. Accordingly, it has been shown that resistant breast cancer cells have higher HER2 expression than endocrine therapy-sensitive cells [39]. Our data perfectly fit in this scenario, suggesting that resveratrol treatment mirrors endocrine resistance acquirement, triggering HER2 up-regulation and ERα repression. Our results also support the hypothesis that resveratrol may exert its anti-ERα action by impairing proteasome-mediated ERα activation. Interestingly, UPS has been described as necessary for a correct activation of ERα [40] and some proteasome inhibitors have been shown to repress ERα gene expression [41, 42]. Moreover, a recently published work has proposed that mTORC1 inhibition activates the Ubiquitin/Proteasome System (UPS) and autophagy [43], thus sustaining an inverse correlation between mTOR signaling and proteasome activity. Consistently, we have reported the resveratrol-induced over-expression of Δ16HER2 eventually results in the activation of mTORC1/p70S6K/p4EBP1 axis. This observation recapitulates a relevant molecular pathway implicated in endocrine resistance development [44] and may explain why the combination of resveratrol and mTORC1 inhibitors have stood out as more effective anti-tumor therapy than single-agent approaches [45, 46].

Interestingly, despite the claimed anti-aging effect of resveratrol, longevity studies have demonstrated that combination of rapamycin and resveratrol was not able to prolong the overall mice survival as mTOR inhibition does [47]. This aspect further strengthens the connection between resveratrol and mTOR pathway and suggests that our data might be extended to a broader context such as aging-related dysfunctions exhibiting a preferential activation of mTORC1 pathway [48].

**Figure 6:**
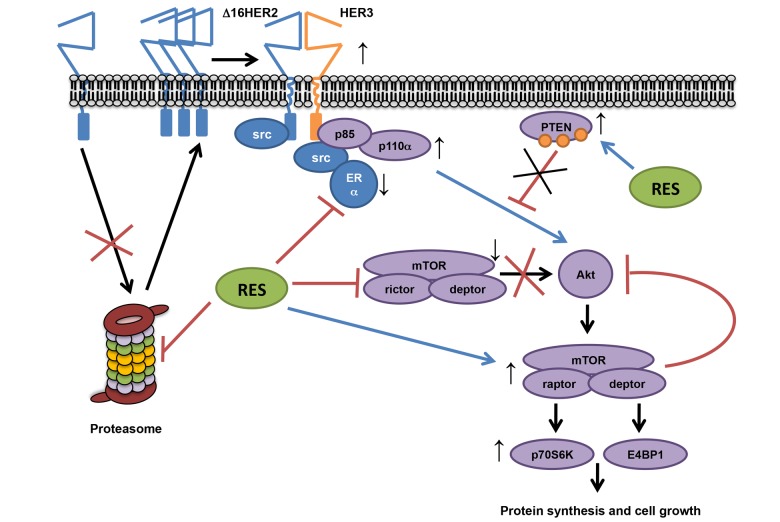
Proposed resveratrol's mechanism of action in a luminal B breast cancer model Our data show that resveratrol down-regulates ERα and lowers the chymotrypsin-like activity of the 20S proteasome in HER2+/ERα+ breast cancer, leading to an increased accumulation of Δ16HER2, which efficiently couples to HER3 and activates the PI3K-AKT-mTOR pathway. In particular, Δ16HER2/HER3 heterodimers trigger the mTORC1/p70S6K/4EBP1 signaling axis inducing an up-regulation of protein synthesis and cell growth. On the other hand, resveratrol inhibits mTORC2 and promotes phosphorylation of PTEN, reducing its catalytic activity, thereby enhancing PI3K-mediated AKT activation, while feedback loops compensate it.

In conclusion, this study provides evidence that the effects of resveratrol treatment depend on intrinsic molecular cancer properties, exerting a tumor-promoting effect in luminal B breast cancer subtype by acting as proteasome inhibitor and inducing a multi-target molecular rearrangement (Figure 6).

### MATERIALS AND METHODS

#### Animals

Δ16HER2 female mice were housed under controlled temperature (20°C) and circadian cycle (12 hours light/12 hours dark) in the animal facility of University of Camerino. The animals were fed on chow diet and tap water *ad libitum*.

#### Cell culture

CAM6 cells, a Δ16HER2-expressing epithelial tumor cell line derived from a mammary carcinoma spontaneously arisen in a Δ16HER2 female [17] and BT474 cell line were maintained in DMEM (Lonza) supplemented with 10% FBS (Gibco, Life Technologies) and 1% penicillin-streptomycin (Gibco, Life Technologies). Cells were cultured at 37°C under humidified atmosphere with 5% CO2.

#### Resveratrol treatment

8 week-old Δ16HER2 female mice were randomly divided into two experimental groups (n=9 each). One group underwent resveratrol treatment and the other was given control vehicle, as previously reported [12]. Briefly, resveratrol (Sigma) was dissolved in methanol to be then diluted in tap water to a final concentration of 1mg/l. Being the daily water intake of about 4 ml/mouse, 4 μg resveratrol/mouse was the daily dose assumed. The control mice were supplemented with 0.02% methanol in tap water (vehicle). Both vehicle- and resveratrol-containing water was renewed twice a week. During the treatment (from 8-weeks to 23-weeks of age), mice were weekly monitored for mammary tumor development by palpation and growing masses greater than 2 mm in mean diameter were regarded as tumors. Two perpendicular diameters (a and b) were measured on each tumor using caliper and volumes were calculated by the V = π/6[(a+b)/2]^3^ formula. All animal experiments were carried out in accordance with the U.K. Animals (Scientific Procedures) Act, 1986 and associated guidelines, EU Directive 2010/63/EU for animal experiments, and were approved by the Ethic Committee on Animal Use of the University of Camerino (protocol number 14/2012). At the end of experiment mice were euthanized and tumors and lungs samples were collected for subsequent analyses. For the *in vitro* experiments resveratrol was dissolved in DMSO (Sigma) and then diluted in complete DMEM to usage concentrations (10-100 μM). 17β-estradiol (Calbiochem) was used as control estrogen treatment (stock solution 40 mM in 100% ethanol and diluted in complete DMEM to 10 nM), as previously described [41]. Cells were treated for 24 hours with resveratrol/17β-estradiol- or vehicle-containing medium (0.02% DMSO).

#### Cell viability assay

Resveratrol effect on cell viability was evaluated by seeding 4×10^4^ cells/well (CAM6 cells) or 2.5×10^4^ cells/well (BT474 cells) in 96-well plates in complete DMEM. The day after, fresh medium containing appropriate resveratrol concentrations was added. Cell viability was determined via MTT assay. MTT (3-(4,5-dimethylthiazol-2-yl)-2,5-diphenyltetrazolium bromide) was purchased from Sigma Aldrich (St. Louis, MO).

#### Immunohistochemistry (IHC) and confocal microscopy analyses

Tumors and lungs harvested from mice were fixed in 4% paraformaldehyde solution for 24-48 hours at room temperature and then embedded in paraffin. Paraffin-embedded sections were stained with hematoxylin and eosin or immunostained with anti-mouse PCNA monoclonal antibody (Dako) or with anti-cleaved caspase-3 monoclonal antibody (MAB835, R&D Systems, Milan, Italy). After washing, they were overlaid with appropriate secondary antibody. Immunostaining was developed with Vulcan Fast Red (Biocare) alkaline phosphatase method. The number of cleaved caspase-3 positive cells was evaluated on digital images of controls and resveratrol tumors (10 per group, 5 × 400 microscopic fields per tumor). As it regards confocal microscopy analysis, after incubation in blocking buffer for 20 minutes, tissue sections were incubated for 1 hour at 37°C with anti-NEU polyclonal antibody (diluted 1:50, Santa Cruz). Nuclei staining was performed using DRAQ5 probe (Life technologies) according to the manufacturer instructions. After washing, the coverslips were incubated with Alexa 488-conjugated goat anti-rabbit secondary antibody (diluted 1:100, Life technologies) for 1 hour at 37°C. Slides were viewed using a Zeiss LSM 510 Meta Confocal Microscope. Quantitative measures were obtained via ImageJ software.

#### Protein extraction and western blot analysis

Tumors and treated cells were homogenized in RIPA buffer (0.1% SDS, 1% NP40, 0.5% CHAPS) supplemented with protease inhibitors aprotinin, sodium orthovanadate and phenylmehylsulfonyl fluoride (Sigma-Aldrich, St. Louis, MO). After 30 minutes’ incubation on ice, whole tumor lysates were centrifuged at 14000 rpm, 4°C, for 20 minutes. The supernatant was collected and proteins were quantified via Bradford assay (Bio-Rad). For western blot analysis equal amounts of protein lysates were separated onto Criterion™ TGX™ precast gels (Bio-Rad) and transferred to a polyvinylidene difluoride (PVDF) membrane (Millipore) using Criterion™ Blotter (Bio-Rad). After blocking in 5% BSA-TBS-T for 1 hour, membranes were incubated with primary antibodies at 4°C overnight. Secondary antibody-binding was performed at room temperature for 1 hour. After TBS-T washing, protein bands were incubated with LiteAblot PLUS reagent (Euroclone) and detected via ChemiDoc™ XRS+ System (Bio-Rad). Densitometry analysis was performed through ImageJ software. Detailed information about the used antibodies is available in Table S1, Supplementary Information.

#### Co-immunoprecipitation

300 μg of tumor cell lysates were incubated overnight with anti-NEU polyclonal antibody (diluted 1:200, Santa Cruz) at 4°C. After washing in RIPA buffer at 2500 g, 4°C for 5 minutes, 200 μl of protein G Sepharose 4 Fast Flow beads (GE Healthcare) were added to each tube. Incubation was performed for 4 hours at 4°C under gentle rocking. Following washing procedure, the samples were boiled in Laemli loading buffer for 5 minutes: the collected supernatants under-went western blot procedure.

#### mRNA extraction, RT-PCR and qRT-PCR

Total RNA was extracted from liquid nitrogen cryo-preserved tumors via TRIzol® reagent (Life Technologies) following the manufacturer instructions. RNA was quantified by measuring 260 nm absorbance via NanoDrop 1000 spectrophotometer (Thermo Scientific). RNA purity was considered good with A260/A280 ratio ≥ 2.0 and A260/A230 ratio ≥ 1.7. RNA (2 μg/tube) was reverse-transcribed using the High-Capacity cDNA Reverse Transcription Kit (Applied Biosystems). Semi-quantitative RT-PCR was performed using Perfect Taq DNA polymerase (5-PRIME) and Eppendorf thermal Cycler. SYBR Premix Ex Taq (Tli RNaseH Plus) reagent (Takara) was used for qRT-PCR analysis. β-actin was taken as housekeeping gene and standard curves for target genes and β-actin were included to evaluate reaction efficiency. Experiments were performed in triplicates. A 2-steps amplification program was carried out on Bio-Rad iCycler Thermal Cycler with iQ5 Multicolor Real-Time PCR Detection System. Primers used: Δ16HER2 forward: 5′-CACCCACTCCCCTCTGAC-3′; Δ16HER2 reverse: 5′-GCTCCACCAGCTCCGTTTCCTG-3′; β-actin forward: 5′-CAAGGCCAACCGCGAGAAGAT-3′; β-actin reverse: 5′-GTCCCGGCCAGCCAGGTCCAG-3′.

#### Proteasome activity

The chymotrypsin-like activity of the 20S proteasome was assayed on tumor and cell extracts using the fluorogenic substrate N-Succinyl-Leu-Leu-Val-Tyr-7-Amido-4-Methylcoumarin (sLLVY-NH-Mec, Sigma-Aldrich). Briefly, tissues/cells were homogenized on ice via a Potter-Elvehjem apparatus in a buffer consisting of 50 mM HEPES/KOH pH 7.8, 1 mM Dithiothreitol and 0.25 M Sucrose. The lysates were then cleared by centrifugation at 12.000 rpm for 10 minutes in a refrigerated Eppendorf centrifuge. Twenty and 40 μg of total proteins from each tissue sample were incubated at 37°C in 100 mM HEPES/KOH buffer, pH 7.8, 5 mM MgCl_2_ and 10 mM KCl. Cell extracts (10 μg) were preincubated for 30 min at 37°C in the same buffer with the addition of different concentrations of resveratrol or with only the vehicle (DMSO). In both cases the reaction was initiated by addition of the fluorigenic substrate to a final concentration of 0.2 mM. The breakdown of the peptide was monitored for 30/45 min using a fluorescence microplate reader (FLUOstar OPTIMA, BMG Labtech GmbH, Offenburg, Germany) with an excitation wavelength of 355 nm and an emission wavelength of 460 nm. Proteasome activity in each sample, expressed as fluorimetric units min^-1^ mg^-1^, was calculated by submitting data to linear regression analysis (R^2^ >0.99).

#### Statistical analysis

Quantitative data are presented as means ± SEM from three independent experiments. The significance of differences was evaluated with two-tailed Students t-test, or one-way ANOVA followed by Bonferroni post-test. Statistical analysis was carried out with GraphPad Prism5.

## SUPPLEMENTAL MATERIAL FIGURES AND TABLES


