# Supplementary material for: Resveratrol fuels HER2 and ERα-positive breast cancer behaving as proteasome inhibitor
Source: Aging (Albany NY). 2017 Feb 26;9(2):508–20. doi: 10.18632/aging.101175 (PMC5361678; doi:10.18632/aging.101175)
Supplement: Supplementary file 1 [file aging-09-508-s001.pdf]

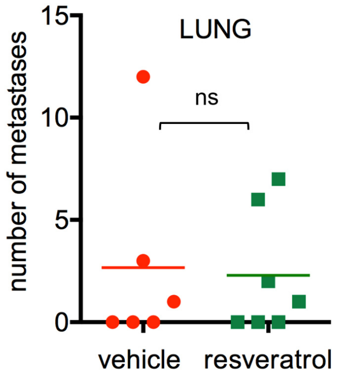

Supplementary Figure 1. Quantization of lung metastases from control and resveratrol supplemented mice. The data presented are the number of lung metastases observed in individual mice, killed at 23-weeks of age (experimental end point). The horizontal bars represent the median value for each group (p=0.8628).

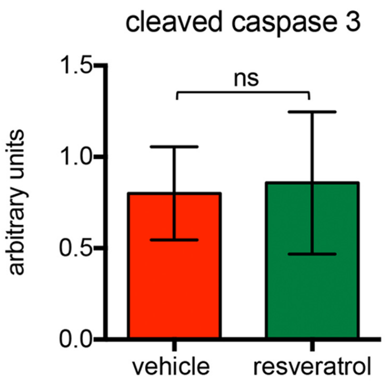

Supplementary Figure 2. Analysis of apoptotic cells in control and resveratrol treated tumors. Quantification of cleaved caspase-3 positive cells was determined by IHC as described in the experimental section. Results are represented as means ± SEM from 5×400 microscopic fields per tumor (n=10). Each group was compared to control (p=0.9134, unpaired two-tailed t test).

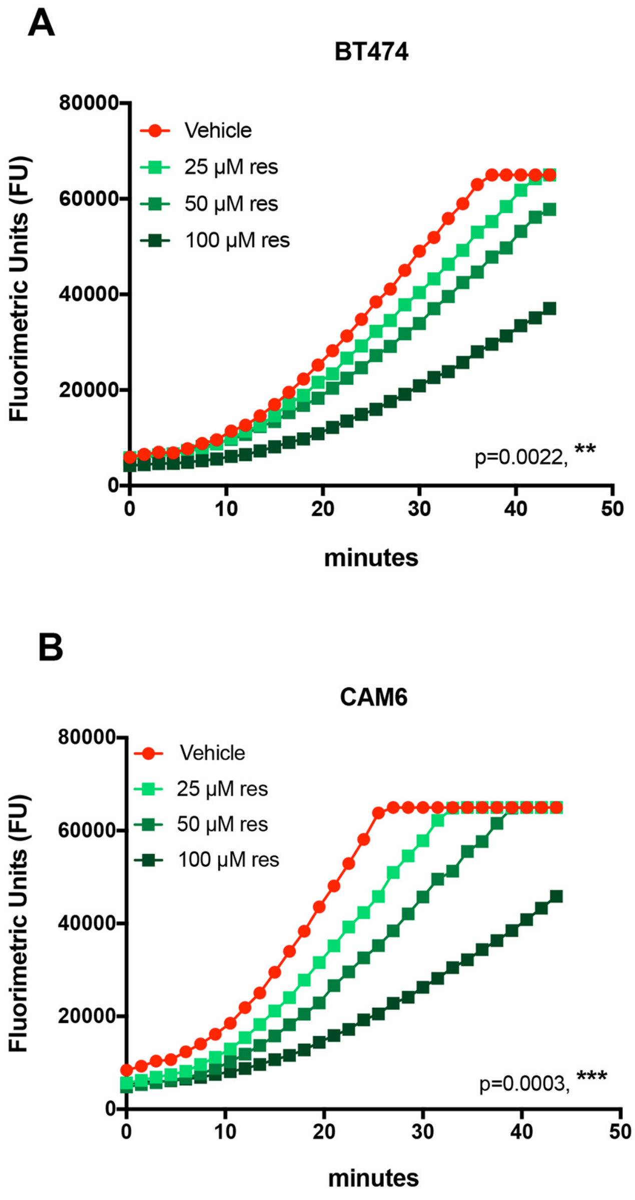

Supplementary Figure 3. Resveratrol inhibits the activity of the 20S proteasome in CAM6 and BT474 cell-free extracts. Cell extracts were pre-incubated with different concentrations of resveratrol or with the vehicle DMSO. The chymotrypsin-like activity of the 20S proteasome was measured in the extracts by addition of the fluorigenic peptide as described in Materials and Methods and expressed as fluorimetric units (FU) min^{-1} mg^{-1}. Statistics: one-way ANOVA

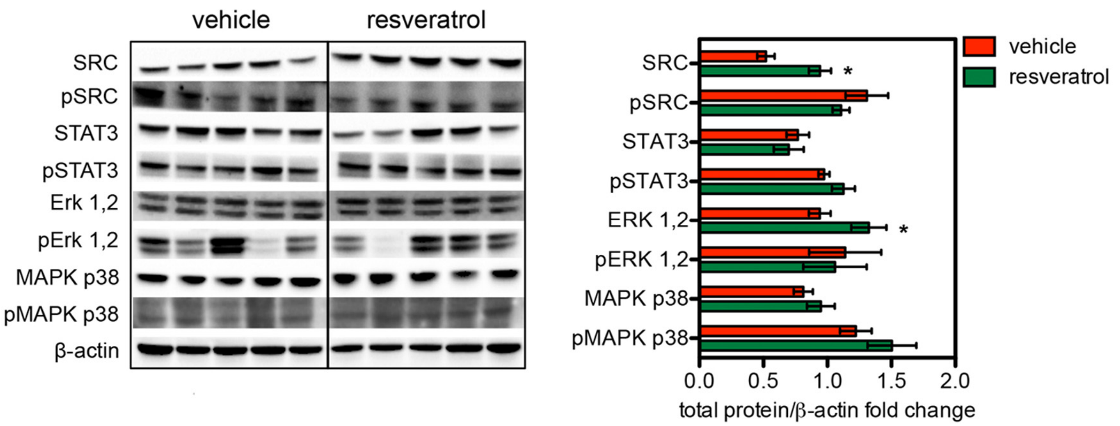

Supplementary Figure 4. Resveratrol supplementation does not activate the Δ16HER2/Src/STAT3 axis. Representative western blot analysis of Δ16HER2 downstream signaling pathways in spontaneous mammary tumors from Δ16HER2 mice, treated or not with resveratrol (left panel), and densitometry quantification from three independent experiments (right panel). Tumor extracts were probed with antibodies to Src, pSrc, STAT3, pSTAT3, Erk, pErk, MAPK p38, pMAPK p38 and β-actin (loading control). The significance was determined by t-test (*$p < 0.05$).

Supplementary Table 1. List of the used antibodies.

PRIMARY ANTIBODIES				
**Antigen**	**Antibody**	**Application**	**Dilution**	**Brand**
**Phospho-HER2**	rabbit monoclonal anti-phospho HER2/ErbB2 (Tyr1248)	**WB**	1:1000	**Cell Signaling Technology**
**HER2**	rabbit monoclonal anti-HER2/ErbB2			
	rabbit polyclonal anti-neu	**IF**	1:50	**Santa Cruz**
**P53**	rabbit polyclonal anti-p53	**WB**	1:1000	
**Phospho-SRC**	rabbit monoclonal anti-phospho SRC (Tyr416)	**WB**	1:1000	**Cell Signaling Technology**
**SRC**	rabbit monoclonal anti-SRC			
**Phospho-STAT3**	mouse monoclonal anti-phospho STAT3 (Tyr705)			
**STAT3**	rabbit monoclonal anti-STAT3			
**Phospho-MAPK p38**	rabbit monoclonal anti-phospho MAPK p38 (Thr 180/Tyr 182)			
**MAPK p38**	rabbit monoclonal Anti-P38			
**Phospho-ERK 1,2**	rabbit monoclonal anti-phospho ERK1/2 p44/42 (Thr202/Tyr204)			
**ERK 1,2**	rabbit monoclonal anti- ERK1/2 p44/42			
**Phospho-AKT**	rabbit monoclonal anti-phospho AKT (Ser473)			
**AKT**	rabbit monoclonal anti-AKT			
**P110α PI3k**	rabbit monoclonal anti-p110α			
**β-actin**	rabbit monoclonal anti-β-actin			
**PTEN**	rabbit monoclonal anti-PTEN			
**Phospho-PTEN**	rabbit monoclonal anti-phospho PTEN (Ser 380, Thr 382, Thr 383)			
**HER3**	rabbit monoclonal anti-HER3			
**Phospho-HER3**	rabbit monoclonal anti-phospho HER3 (Tyr1289)			
**mTOR**	rabbit monoclonal anti- mTOR			

**Phospho-mTOR**	rabbit monoclonal anti-phospho mTOR (Ser 2448)			
	rabbit monoclonal anti-phospho mTOR (Ser 2481)			
**Rictor**	rabbit monoclonal anti-rictor			
**Phospho-p70S6K**	rabbit monoclonal anti-phospho p70S6K (Thr 389)			
**Phospho-E4BP1**	rabbit monoclonal anti-phospho E4BP1 (Thr37/46)			
**Raptor**	rabbit monoclonal anti-raptor			
**P85-PI3k**	rabbit monoclonal anti-p85	**WB**	1:1000	**Epitomics**
**ERα**	mouse monoclonal anti-ERα	**WB**	1:1000	**ORIGENE**
**PCNA**	rabbit monoclonal anti-PCNA	**IHC**	1:200	**Dako**
**Cleaved caspase-3**	rabbit anti-cleaved caspase-3	**IHC**	1:30	**R&D Systems**
**20S Proteasome**	rabbit polyclonal anti 20S subunits	**WB**	1:1000	**Enzo Life Science**
**Ubiquitin**	rabbit polyclonal anti ubiquitin	**WB**	1:1000	**kindly provided by Prof. AL Haas**

**SECONDARY ANTIBODIES**			
**Antibody**	**Application**	**Dilution**	**Brand**
**HRP-conjugated goat anti-mouse IgG (H&L)**	**WB**	1:3000	**Calbiochem**
**HRP-conjugated goat anti-rabbit IgG (H&L)**	**WB**	1:20000	**Sigma-Aldrich**
**Biotin-conjugated goat anti-rabbit IgG (H&L)**	**IHC**	1:200	**Bethyl Laboratories**
**Alexa Fluor® 488-conjugated goat anti-rabbit IgG (H&L)**	**IF**	1:100	**Life Technologies**
